# Minimal data requirement for realistic endoscopic image generation with Stable Diffusion

**DOI:** 10.1007/s11548-023-03030-w

**Published:** 2023-11-07

**Authors:** Joanna Kaleta, Diego Dall’Alba, Szymon Płotka, Przemysław Korzeniowski

**Affiliations:** 1 Sano Centre for Computational Medicine, Krakow, Poland; 2 Department of Computer Science, University of Verona, Verona, Italy; 3 Informatics Institute, University of Amsterdam, Amsterdam, The Netherlands; 4 Department of Biomedical Engineering and Physics, Amsterdam University Medical Center, Amsterdam, The Netherlands

**Keywords:** Diffusion models, Synthetic data generation, Surgical simulation

## Abstract

**Purpose:**

Computer-assisted surgical systems provide support information to the surgeon, which can improve the execution and overall outcome of the procedure. These systems are based on deep learning models that are trained on complex and challenging-to-annotate data. Generating synthetic data can overcome these limitations, but it is necessary to reduce the domain gap between real and synthetic data.

**Methods:**

We propose a method for image-to-image translation based on a Stable Diffusion model, which generates realistic images starting from synthetic data. Compared to previous works, the proposed method is better suited for clinical application as it requires a much smaller amount of input data and allows finer control over the generation of details by introducing different variants of supporting control networks.

**Results:**

The proposed method is applied in the context of laparoscopic cholecystectomy, using synthetic and real data from public datasets. It achieves a mean Intersection over Union of 69.76%, significantly improving the baseline results (69.76 vs. 42.21%).

**Conclusions:**

The proposed method for translating synthetic images into images with realistic characteristics will enable the training of deep learning methods that can generalize optimally to real-world contexts, thereby improving computer-assisted intervention guidance systems.

## Introduction

Computer-assisted intervention (CAI) is a research field focused on enhancing the safety, efficiency, and cost-effectiveness of medical procedures by minimizing errors and complications [15]. Within CAI, laparoscopic cholecystectomy (LC) has gained significant attention as a widely performed minimally invasive procedure for gallbladder removal [19]. However, LC presents technical challenges due to limited visibility and the use of laparoscopic instruments, leading to potential complications like bile duct injury (BDI) [31]. To address these complexities, CAI systems leveraging deep learning (DL) methods have been proposed. These systems aim to identify safe dissection zones, locate anatomical landmarks, and automatically assess critical safety criteria [14, 31]. DL techniques, including action triplet recognition, temporal modeling, tools segmentation, and segmentation of anatomical structures, have been applied to LC [7, 19, 32, 35].

However, the availability of annotated data poses challenges for training DL models in this domain [22]. Limited datasets, primarily derived from the Cholec80 dataset, exist for LC, annotated with phases, tool presence, and action triplets [18, 19, 32]. To overcome this limitation, generating synthetic data through virtual simulations along with rich annotations has been explored [4, 22]. Yet, DL models trained on synthetic data often struggle to perform well on real data due to the domain gap [22]. Image-to-image translation techniques based on generative adversarial networks (GANs) have been proposed to mitigate this limitation [2]. However, these techniques still require a substantial amount of annotated data.

Recently, latent diffusion models (LDMs) have shown promise in generating highly detailed images while preserving semantic structure [9]. LDMs employ an iterative process involving noise addition and reverse learning to recover original data. In the medical field, LDMs have been extensively utilized for tasks such as image translation, generation, preprocessing, segmentation, and classification [9]. Compared to other DL techniques like GANs, LDMs can be fine-tuned effectively with smaller datasets and combined with support methods for controlled generation. The widely used Stable Diffusion (SD) LDM model offers efficient conditioning of the generation process through text prompts [24].

**Fig. 1:**
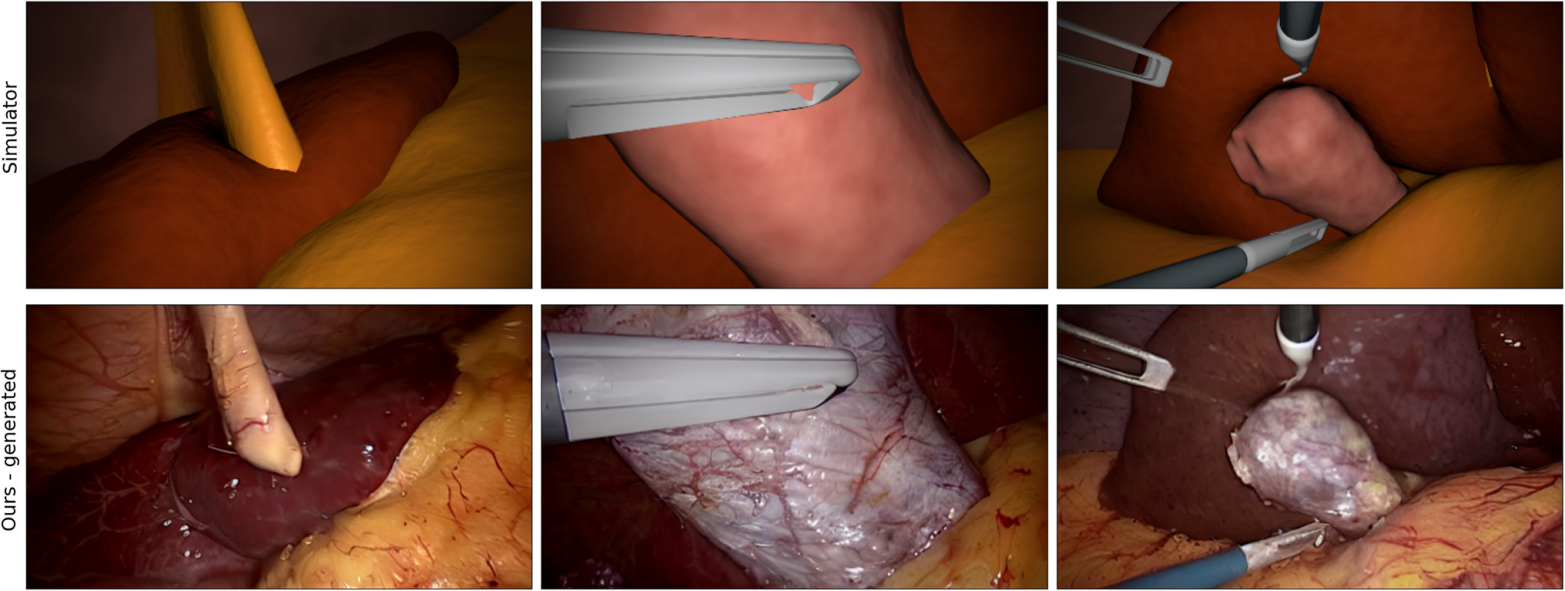
Examples of synthetic data translated with our fine-tuned model to the CholecT45 style are shown. Three random frames from the simulator and their realistic translations are shown in the top and bottom rows, respectively

In general, no existing work uses LDMs instead of GANs for the translation of synthetic images into realistic images. Therefore, our contributions are as follows:

(1) We introduce a novel application of the Stable Diffusion model to generate synthetic surgical data in an unsupervised manner, addressing the issue of limited data availability in clinical environments, see Fig. 1. To the best of our knowledge, this approach has not been previously published. (2) We evaluate our approach using public datasets to demonstrate its effectiveness in generating realistic synthetic data. The results show that our approach outperforms the baseline method in preserving tissue integrity, achieving a mean Intersection over Union (mIoU) of 69.76% compared to 42.21% for the CholecT80-style baseline. Additionally, our method successfully captures the characteristic feature distribution of real surgical data, either comparable to or enhanced compared to the baseline dataset. (3) We provide public access to the code and our realistic rendering of the publicly available IRCAD dataset, which includes simulation frames, depth maps, segmentation maps, edges, and normals at https://github.com/SanoScience/sim2real_with_Stable_Diffusion.

## Related work

Several approaches have been proposed for generating synthetic data with realistic characteristics for specific surgical procedures, e.g., [10]. In one study [4], Unity3D was used to create a 3D liver and laparoscope environment, enabling the generation of images for DL segmentation training. Another study [16] employed a GAN approach to directly generate images from segmentation maps, focusing on maximizing differences between instruments and anatomical environments. The combination of synthetic images and real segmentation maps has been extensively used to train GANs for surgical tool segmentation, employing techniques like consistency losses and student–teacher learning [26, 27]. GAN-based approaches have also been applied in other surgical domains such as cardiac intervention, colonoscopy examination, and sinus surgery [13, 20, 29]. While numerous other GAN-related works exist, they are beyond the scope of this discussion [2].

Another relevant work [22] introduced an image-to-image translation method for simplified 3D rendering of LC anatomy based on real endoscopic images from the Cholec80 dataset. This approach utilized GANs trained in an unpaired manner and generated a dataset of 100,000 images with various annotations. Although extended to video translation [23], this work uses only simplified liver views. It lacks surgical tools and the specific anatomy of interest (gallbladder), making it not representative of LC procedures.

While GAN-based approaches have shown potential, they have limitations, such as early convergence of discriminators and instability of the adversarial loss function, which can lead to mode collapse and reduced diversity in generated data. Diffusion models (DMs) have emerged as a promising alternative, surpassing GANs in computer vision tasks [3]. In the medical domain, DMs have been widely utilized for various applications, including generating MRI sequences, synthesizing histological images, and generating thoracic X-ray images based on text prompts [9, 17, 21]. The latter employs the SD model, which uses text prompts as conditioning and has been applied successfully in similar medical tasks. Notably, this is the first work to utilize DMs for generating intra-operative endoscopic images, combining text prompts with virtual simulator images for conditioning the process.

## Methods

**Fig. 2:**
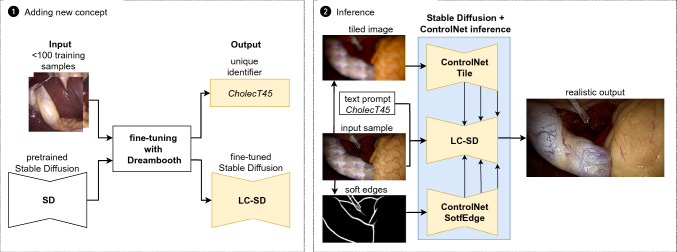
Overview of fine-tuning and inference: SD is fine-tuned using DreamBooth, which binds a unique text identifier with a newly incorporated CholecT45 style. In the inference stage, the fine-tuned LC-SD is conditioned with two ControlNet models. The textured input sample, along with the ’cholecT45’ prompt, is passed to LC-SD. The Tile ControlNet accepts a tiled version of the input sample, while the SoftEdge ControlNet accepts edges detected by Pidinet from the input sample

Our method involves adding a concept to the SD model and using it to generate realistic images from synthetic ones. We begin by fine-tuning SD based on DreamBooth (DB) [25]. Then, the fine-tuned Laparoscopic Cholecystectomy Stable Diffusion (LC-SD) model is employed to generate realistic images. This is achieved by leveraging two versions of the ControlNet support architecture, namely Tile and SoftEdge control, to ensure consistency between label and generated images. An overview of the proposed method is depicted in Fig. 2.

### Fine-tuning with DreamBooth

SD [24] is a LDM that implements a denoising technique in a lower-dimensional latent space. One of the key features of SD is its flexibility in conditioning the denoising step on various modalities, such as text or images, achieved through a cross-attention mechanism. Significant progress has been made in the field of SD model few-shot fine-tuning and personalized concept introduction through notable works, including Textual Inversion [5], Low-Rank Adaptation [8], Custom Diffusion [11], and DB [25]. Among these, DB has been selected as it utilizes a fine-tuning approach with a small set of concept-specific images (3 to 5 for an object and 50 to 200 for a style) and allows for the introduction of highly unconventional concepts. During training, the model is paired with text prompts containing class names and unique text identifiers. The model learns to associate the text identifier with the new concept. By incorporating a class-specific prior preservation loss, DB encourages the generation of diverse instances within the subject’s class, resulting in the synthesis of photorealistic images.

DB has a tendency to overfit, and the appropriate number of training steps depends on several factors, including the characteristics of the training data, learning rate, and prior preservation. This number needs to be determined experimentally. In our specific case, we did not use prior preservation during fine-tuning because SD lacks a proper prior for human tissue or surgical images. Additionally, unlike the original work where the text encoder was frozen, we fine-tuned both the text encoder and the U-Net.

### Inference with ControlNet

To generate realistic tissues based on simulation scenes, we employ text-guided image-to-image inference. During the inference stage, we utilize a unique text identifier that was bound with the CholecT45 style during DB training.

ControlNet is an architecture designed to control pretrained large DMs by incorporating additional conditions, such as sketches, key points, edge, and segmentation maps [36]. It maintains two sets of U-Net weights copied from pretrained DM: a trainable copy and a locked copy. The locked copy preserves the original weights from the pretrained DM during training. The trainable copy is fine-tuned using task-specific datasets to adapt to the additional conditions and introduces control during inference. Neural network blocks of pretrained DM and ControlNet model are connected with the use of trainable “zero convolution” layers which parameters are optimized during ControlNet training. Explanation of “zero convolution” layers function, block connection and application of ControlNet to the original SD are described in detail in [36].

Since our method operates in a limited real data setting, training a custom ControlNet is not feasible as it would require at least a few thousand diversified images along with conditioning inputs. However, it is possible to directly apply ControlNet trained on original SD to LC-SD and even to combine multiple ControlNet models (each with desired strength) to impose diversified control. To combine single LC_SD block with corresponding blocks of *N* ControlNets, we present the extended formula from [36] as:

1 $$\begin{aligned} {\varvec{y}}_{\text {c}}= & {} {} {\mathcal {F}} ({\varvec{x}}; \varvec{\theta }_{LC\_SD}) + \nonumber \\{} & {} + \sum _{i=1}^{N} w_i {\mathcal {Z}} \left( {\mathcal {F}}\left( {\varvec{x}} + {\mathcal {Z}}\left( {\varvec{c}}_i; \varvec{\theta }_{Z1, i}\right) ; \varvec{\theta }_{C, i}\right) ; \varvec{\theta }_{Z2, i}\right) \end{aligned}$$

where ${\varvec{x}}$ is an input feature map to the LC-SD block, ${\varvec{c}}_i$ is a conditioning input feature map to the corresponding block of the *i*th trained ControlNet, and ${\varvec{y}}_{\textrm{c}}$ is a conditioned output feature map from the LC-SD block. We denote the weights of the LC-SD block as $\varvec{{\theta }_{LC\_SD}}$ and the trainable weights for the block of the *i*th ControlNet as $\varvec{\theta _{C,i}}$. The function denoted as $\mathcal {F(\cdot ; \cdot )}$ transforms the input feature map into the output feature map given a set of parameters. We denote the “zero convolution” operation as $\mathcal {Z(\cdot ; \cdot )}$. Within the block of the *i*th ControlNet, two “zero convolution” operations are performed with optimized parameters $\{\varvec{\theta }_{Z1, i}, \varvec{\theta }_{Z2, i}\}$, respectively. $w_i$ is the strength the *i*th ControlNet is applied with. The first term on the right side of Eq. 1 represents the result of applying LC-SD, while the second term relates to the contribution of the different ControlNets.

Given the variety of available pretrained ControlNets [36], we explored additional outputs from the simulator as potential control inputs. However, the Segmentation ControlNet was not applicable since it requires a segmentation map compliant with ADE20K’s segmentation format, which does not include any surgical-relevant class/label. We also conducted preliminary tests on depth and normal ControlNets using our inference IRCAD dataset, which is described in detail in Sect. "Dataset and implementation details". While both control inputs helped maintain proper anatomical boundaries, the depth details were not captured, and the overall visual performance was unsatisfactory.

Instead, we focused on control methods that could be robustly applied to completely new styles: **SoftEdge v1.1** and **Tile v1.1**. Both ControlNets contribute to generating consistent shapes and boundaries, but they impose additional constraints on different aspects of the output images. The SoftEdge control utilizes edges generated with Pidinet [30] or HED [34] models. It primarily preserves original edges and tissue folds. On the other hand, Tile ControlNet exhibits conceptual similarities with tile-based super-resolution models but offers broader applications. It operates in two modes: generating new details while ignoring existing ones, and ignoring global prompts when local tile semantics and prompts do not align, guiding the diffusion process with local context. In the context of endoscopic image generation, Tile ControlNet effectively adds tissue details and helps preserve accurate tissue colors.

## Experiments

### Dataset and implementation details

To use the minimum amount of data while ensuring a sufficient variability of visual properties and the presence of all regions and instruments of interest, we trained three separate models, each based on two distinct videos from the CholecT45 dataset [19]. We carefully select pairs of videos that exhibit comparable visual characteristics and ensure that all classes are represented within each training set. We train each model with DB using a manually selected set of 85, 91, and 95 images, respectively. Despite the limited number of images, it is crucial to choose representative and consistent samples that cover various procedure stages and tissues present in the synthetic dataset. Furthermore, to prevent the models from introducing tool artifacts in each frame, it was highly important to include images both with surgical tools and with minimal or no presence of them. All models are based on Stable Diffusion v1.5, and we train them with DB using a learning rate of 1 $\times$ $10^{-6}$ and a batch size of 4 for 2,000 steps.

**Fig. 3:**
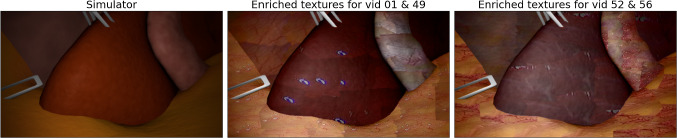
Visual comparison between the raw image from the simulator (first from left) and image with enriched textures for two styles (second and third from left)

**Fig. 4:**
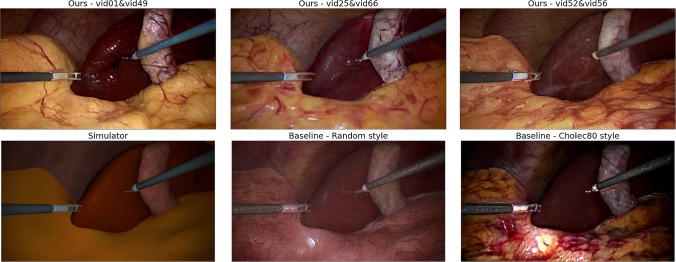
A visual comparison is made between the data processed using our fine-tuned Stable Diffusion model (first row), the raw image from the simulator, and the data generated in [22] for random and Cholec80 styles (second row)

In the inference stage, we utilize fully labeled synthetic data from the IRCAD 3D CT liver dataset, as previously employed in [22]. The dataset contains 20,000 synthetic images rendered from 3D scenes obtained from the CT data of 10 different patients including models of the liver, gallbladder (only for 6 patients), insufflated abdominal wall, fat, and connective tissue. In addition, tools, light sources, and endoscopic cameras have been added in random positions in the scene. In the image-to-image approach, the prior information significantly influences the resulting image. However, the IRCAD dataset presents simplified anatomy, and as a result, plain structures and distorted colors can lead to unrealistic results. To address this issue, we enhance the raw simulation images by incorporating texture information from example samples. We extract small texture samples for each tissue from the corresponding training set and blend them with the raw simulation scenes, guided by segmentation maps, as shown in Fig. 3.

**Table 1:** Selected inference parameter values for each model: denoising strength, CFG, noise scheduler, SoftEdge, and Tile control strength. All the models use noise scheduler DPM++ 2 M Karras

Style	Denoising	CFG	SoftEdge	Tile
CholecT45 vid52 & vid56	0.45	4.5	0.5	0.3
CholecT45 vid25 & vid66	0.45	5.0	0.4	0.3
CholecT45 vid01 & vid49	0.5	5.0	0.55	0.3

**Fig. 5:**
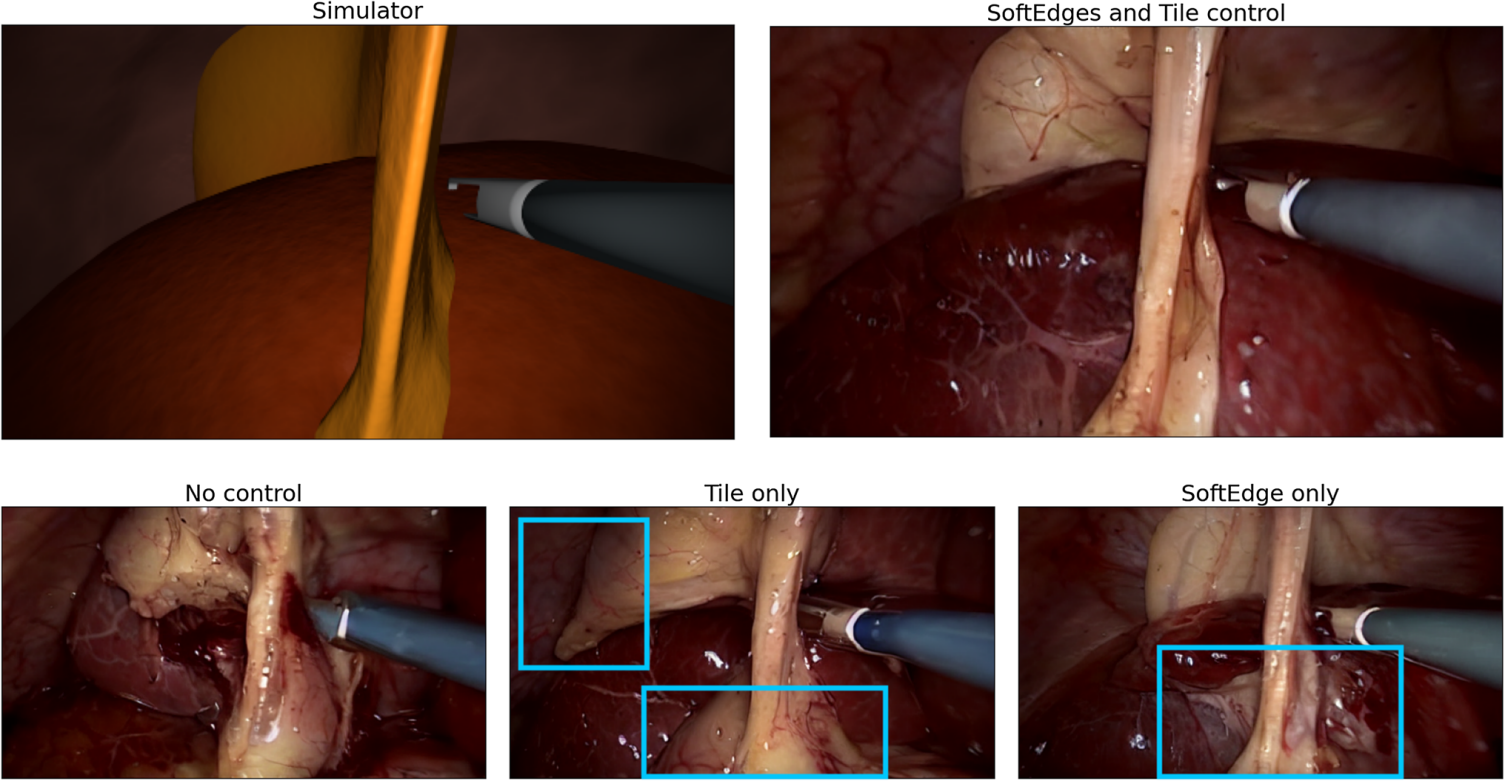
Visual comparison on example image generated with different control types applied. Without any type of control, the overall image consistency is degraded. With Tile control details are clearly rendered, but tissue shapes do not correspond to the given labels. With only SoftEdge control color artifacts appeared

For inference, we adjust the model checkpoint, denoising strength, classifier-free guidance scale (CFG), noise scheduler, and ControlNet strengths for each LC-SD model separately. Although all LC-SD models are trained with the same parameters, variations in the complexity and diversity of the training sets resulted in differences in denoising capabilities across the models. We carefully balance the ControlNet strengths for each model separately. In addition to tissue placement, we also consider overall image realism and details, such as tissue folds. The lack of tissue folds would not necessarily degrade mIoU. To achieve the desired balance, we use a stronger SoftEdge control in combination with a weaker Tile control. Using only SoftEdge with high control strength could compromise image quality by erasing valuable details. To prevent Tile control from introducing excessive detail based on the input sample, we use a smaller strength. To generate data at a large scale while maintaining reasonable inference time and acceptable image quality, we limit the denoising steps to 20. The selected parameter values are shown in Table 1.

The data generation process is carried out on a single NVIDIA A100 GPU. The generation time takes up to 3 s per image, depending on the number of ControlNets utilized.

### Evaluation metrics

To evaluate the realism of the generated data, we employ established evaluation metrics[9, 23]: Frechet Inception Distance (FID) [6] and Kernel Inception Distance (KID) [1]. These metrics assess the similarity between two sets of images based on their feature representations extracted from a pretrained Inception network. Following [12, 33, 38], we employ Learned Perceptual Image Patch Similarity (LPIPS) [37] to assess diversity of generated samples. LIPIS is an image quality assessment method, it computes the average distances between samples in AlexNet, VGG, or Squeezenet feature space.

Moreover, to evaluate the LC-SD models’ ability to preserve labels, we fine-tuned a variant of U-Net with a pretrained ResNet50 backbone using the CholecSeg8k dataset [7] for five classes: abdominal wall, liver, fat, gallbladder, and tool. These classes are present in both the CholecSeg8k and IRCAD datasets. From the training data, we exclude videos used to generate synthetic data. We use mean Intersection over Union (mIoU) to calculate the average overlap between predicted and ground truth segmentation masks across multiple classes which allows us to evaluate the models’ ability to preserve labels. The mIoU for the real test data was 89.77%, and we assessed the mIoU for 10,000 generated images.

**Table 2:** Quantitative results are presented for each style: the raw simulator, baseline data, and our generated data. We demonstrate this using mIoU, FID, KID, and LPIPS metrics

Method	Style	mIoU [%] $\uparrow$	FID $\downarrow$	KID $\downarrow$	$\textrm{LPIPS}_{\textrm{VGG}} \uparrow$
N/A	Raw simulation images	24.73	305.00	.3739 ±.0041	.5820
[22]	Random	45.28	110.92	.1243 ±.0035	.5834
[22]	Cholec80	42.21	67.13	.0623 ±.0017	**.6407**
Ours	CholecT45 vid52 & vid56	66.85	68.35	.0658 ±.0015	.6245
Ours	CholecT45 vid25 & vid66	**69**.**76**	63.07	.0582 ±.0012	.6262
Ours	CholecT45 vid01 & vid49	67.20	57.47	.0513 ±.0011	.6175
Ours	Mixed styles	67.89	**54**.**57**	**.0473** ± **.0011**	.6281

The best-performing methods are in bold

**Table 3:** The mIoU [%] values for different control types and improvement compared to no-control inference are presented

Style	No control	Only SoftEgde	Only tile	SoftEdge + Tile
CholecT45 vid52 & vid56	61.52	65.26 ($+$ 6.1%)	64.20 ($+$4.4%)	**66.85 (+8.7%)**
CholecT45 vid25 & vid66	63.35	67.16 ($+$ 6.0%)	68.01 ($+$7.4%)	**69.76 (+10.1%)**
CholecT45 vid01 & vid49	54.29	63.26 ($+$ 16.5%)	62.08 ($+$14.3%)	**67.20 (+23.8%)**

Bold font indicates the best results

## Results

A visual comparison revealed that our generated data achieve similar perceptual realism to the baseline dataset [22], as visible in the samples shown in Fig. 4. Table 2 presents the quantitative results obtained from various methods and styles, with the best scores highlighted in bold. The first row represents raw simulation images with a mIoU of 24.73%, FID of 305.00, KID of 0.3739 ± 0.0041, and LPIPS of 0.5820. The second and third rows, attributed to the method presented in [28], demonstrate improvements in performance. For the random style, the mIoU and LPIPS increase to 45.28% and 0.5834, respectively, while the FID decreases to 110.92 and the KID to 0.1243 ± 0.0035. When using the Cholec80 style, the mIoU remains high at 42.21%, while the FID and KID values drop to 67.13 and 0.0623 ± 0.0017, respectively. The LPIPS obtains the highest result of 0.6407. Subsequently, our method, denoted as “ours,” showcases further enhancements. With the CholecT45 vid52 & vid56 style, we achieve an impressive mIoU of 66.85%, accompanied by an FID of 68.35, a KID of 0.0658 ± 0.0015, and LPIPS of 0.6245. Notably, the best performance is attained when employing the CholecT45 vid25 & vid66 style, achieving a remarkable mIoU score of 69.76%, with an FID of 63.07, a KID of 0.0582 ± 0.0012, and LPIPS of 0.6262. Additional experiments demonstrate the effectiveness of our method with different styles, such as CholecT45 vid01 & vid49, resulting in a mIoU of 67.20%, an FID of 57.47, a KID of 0.0513 ± 0.0011, and LPIPS of 0.6175. Moreover, when applying mixed styles, our approach attains a high mIoU of 67.89%, while achieving the best FID score of 54.57 and KID score of 0.0473 ± 0.0011, while LPIPS is 0.6281. Our method achieves significantly higher mIoU and, depending on the style, either lower or comparable FID, KID, and comparable LPIPS.

Figure 5 and Table 3 provides an overview of the mIoU values for different control types and their improvements compared to no control inference. The table shows that the combined control models yielded the best results overall, with the highest improvement observed for the style vid01 & vid49 when inferred with the highest denoising value. For the CholecT45 vid52 & vid56 style, the no-control inference resulted in a mIoU of 61.52%. When applying only SoftEdge control, the mIoU increased to 65.26 ($+$6.1%), while using only Tile control led to a mIoU of 64.20 ($+$4.4%). The most significant improvement of 8.7% was achieved when combining SoftEdge and Tile controls, resulting in mIoU of 66.85%. Similarly, for the CholecT45 vid25 & vid66 style, the no-control inference yielded a mIoU of 63.35, which increased to 67.16 ($+$6.0%) when using only SoftEdge control and to 68.01 ($+$7.4%) with only Tile control. However, the best performance was obtained when both SoftEdge and Tile controls were combined, resulting in mIoU of 69.76%, representing a substantial improvement of 10.1%. Furthermore, for the CholecT45 vid01 & vid49 style, the no-control inference achieved a mIoU of 54.29%. By employing only SoftEdge control, the mIoU increased to 63.26 ($+$16.5%), while using only Tile control resulted in a mIoU of 62.08 ($+$14.3%). Notably, the highest improvement of 23.8% was attained when combining SoftEdge and Tile controls, leading to a mIoU of 67.20%.

## Discussion and conclusions

In this work, we have proposed an SD-based approach to generate realistic surgical images from virtual simulator images and text prompts. The SD model was initially fine-tuned using DB and then used for inference, supported by Tile and SoftEdge ControlNets. The model can be trained using less than 100 real images without manual annotations and manages to generate realistic images that outperform the baseline in all considered evaluation metrics.

We consider this work to be a significant addition to the current foundation, offering researchers a valuable dataset to facilitate the development of machine learning solutions in image-guided and robotic surgery. This approach can produce fully labeled training data for supervised machine learning algorithms. Additionally, strict alignment of the created data with its ground truth annotations extends its potential for evaluation in various unsupervised and semi-supervised applications.

Despite that, our method has several limitations. Firstly, the use of a very limited training dataset makes image selection critical, requiring careful consideration to ensure representativeness and consistency. Secondly, our method heavily relies on the input image features. We leave addressing this limitations for future work. Temporal consistency is another major limitation of our approach, which we could not investigate in depth due to the lack of temporal coherence in the simulated data. To address this, a more detailed synthetic dataset with enhanced textures and tool–tissue interactions, along with extended annotations, would be necessary to support different tasks, such as surgical temporal modeling.

Overall, our proposed method represents a promising direction for generating realistic surgical images and has the potential to contribute to advancements in the field of image-guided and robotic surgery.
